# Community-based interventions in hypertensive patients: a comparison of three health education strategies

**DOI:** 10.1186/s12889-015-1401-6

**Published:** 2015-01-29

**Authors:** Chu-Hong Lu, Song-Tao Tang, Yi-Xiong Lei, Mian-Qiu Zhang, Wei-Quan Lin, Sen-Hua Ding, Pei-Xi Wang

**Affiliations:** Department of Preventive Medicine, School of Public Health, Guangzhou Medical University, Guangzhou, China; Community Health Services Center of Liaobu, Dongguan, China

**Keywords:** Community-based intervention, Health education, Hypertension, Blood pressure, Serum lipids

## Abstract

**Background:**

Community-based health education programs may be helpful in improving health outcomes in patients with chronic illnesses. This study aimed to evaluate community-based health education strategies in the management of hypertensive patients with low socioeconomic status in Dongguan City, China.

**Methods:**

This was a randomized, non-blinded trial involving 360 hypertensive patients enrolled in the community health service centre of Liaobu Town, Dongguan City, China. Participants were randomized to receive one of the three community-based health education programs over 2 years: self-learning reading (Group 1), monthly regular didactic lecture (Group 2), monthly interactive education workshop (Group 3). Outcomes included the changes in the proportion of subjects with normalized blood pressure (BP), hypertension-related knowledge score, adherence to antihypertensive treatment, lifestyle, body mass index and serum lipids.

**Results:**

After the 2-y intervention, the proportion of subjects with normalized BP increased significantly in Group 2 (from 41.2% to 63.2%, p<0.001), and increased more substantially in Group 3 (from 40.2% to 86.3%, p<0.001), but did not change significantly in Group 1. Improvements in hypertension-related knowledge score, adherence to regular use of medications, appropriate salt intake and regular physical activity were progressively greater from group 1 to group 2 to group 3. Group 3 had the largest reductions in body mass index and serum LDL cholesterol levels.

**Conclusion:**

Interactive education workshops may be the most effective strategy in community-based health promotion education programs for hypertensive patients in improving patients’ knowledge on hypertension and alleviating clinical risk factors for preventing hypertension-related complications.

## Background

Hypertension is a serious public health concern. More than one-quarter of the adult population over the world has hypertension, a significant health burden in many countries [1,2]. As a major chronic non-communicable disease, hypertension is the most important risk factor for cardiovascular and kidney diseases, stroke and premature death if not detected early and treated appropriately. The estimated prevalence of hypertension in China is 22% in Chinese adult population, corresponding to about 200 million hypertensive patients [3,4]. Targeted interventions for patients with hypertension to control blood pressure are needed to improve health related quality of life and reduce hypertension-related complications and mortality in China.

Health education may result in lifestyle modifications and increase adherence to antihypertensive medications to improve effective blood pressure (BP) control in hypertensive patients [4,5]. In general, health education may improve patients’ knowledge on a disease and its therapy leading to better treatment adherence and patients taking a more positive role in the management of their health [6-10]. However, it is unclear what health educational strategy works best in improving patients’ knowledge on hypertension and possibly clinical outcomes in hypertensive patients. There is now an increasing community-based effort in the prevention and control of hypertension in China. Hypertension is a major chronic disease that is often managed at community health service centers in China. Health education may play a key role in the management of hypertensive patients [11,12]. The common tools of health education in community health centers in China include health posters, health booklets, individualized lecture, and public lecture [13]. As a developing country, health education is still in an experimental stage in Chinese communities. There are some limitations in most currently available health education programs in China. The contents in most health education programs are often difficult to understand for lay readers, considering that most patients have relatively low educational levels. The educational methods may be somewhat boring and ineffective [14].

The community health service center in Liaobu Town, Dongguan City in recent years developed an interactive health education workshops program in the management of hypertensive patients. It is one of the national health education demonstration project, and the first one to implement comprehensive health education focusing on patients’ participation through dynamic and interactive workshops. The educational tools include cartoon pictures and animations illustrating cardiovascular disease progress models, treatment and prevention measures. It is designed to accommodate the educational levels of the majority of hypertensive patients (primary or middle school) managed at community health centers in China. The aim of this study was to evaluate this new interactive health education workshops program in comparison with two common health education strategies (self-learning reading, regular didactic lecture) by assessing the changes in hypertension-related knowledge, antihypertensive medications adherence, lifestyle and anthropometric, biochemical and clinical parameters.

## Methods

### Study design

This study was a randomized, non-blinded community-based health education trial involving 360 participants at the Community Health Service Centre in Liaobu Town, Dongguan City, China. The study was approved by the Research Ethics Board of Guangzhou Medical University (Guangzhou, China). Informed consent was obtained from all study participants. The trial was registered at Chinese Clinical Trial Registry (registration number ChiCTR-OPC-14005283). We followed the CONSORT guidelines in reporting RCTs.

### Participants and intervention

Patients were recruited among hypertensive patients managed at the Community Health Service Centre in Liaobu Town, Dongguan City, China. Patients were eligible if they met the following inclusion criteria: a clinical diagnosis of hypertension; conscious (capable of effective oral communication without help); age between 40 and 75 years; completed primary school or higher education; able to communicate with educators; availability to participate in assigned health education activities. Patients were not eligible if they met anyone of the following exclusion criteria: pregnancy; mental disorders, dementia or cognitive impairment; other serious diseases with the need for special care such as malignant tumors, heart failure, kidney disease, AIDS.

The recruitment was conducted in September 2011. With an estimated proportion of normalized blood pressure at 40.0% after 2-y of BP control medications, a sample size of 102 per group is required to detect an improvement to 56.0% in normalized BP after the health education intervention, with an alpha error of 5% and a power of 90%. In our study, a total of 360 eligible patients agreed to participate in the study. They were assigned randomly to one of the three health education on hypertension groups by a statistician who was blinded to the intervention using a computer-generated random sequence number. Group 1 (self-learning reading, n=120) participants received orientation on reading materials to learn knowledge on hypertension through the poster text messages on blackboards and health education booklets monthly. Group 2 (regular lecture, n=120) participated in a public didactic lecture on hypertension monthly by phone invitation. Each lecture lasted about 30 minutes. Group 3 (interactive education workshop, n=120) participated in an interactive participatory education workshop on hypertension knowledge monthly. The interactive education workshop on hypertension was given through the active involvement of participants in the use of visual health education tools (cartoon pictures, animation, food models, salt spoons, oil pots, pedometer and cardiovascular disease models). The number of individuals in group 2 and group 3 shall not be less than 10 in each class. For absentees, the next available lecture would be arranged within a month.

**Figure 1:**
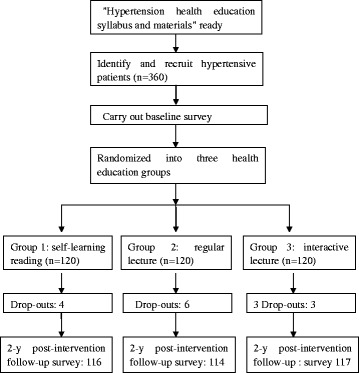
**Flowchart illustrating patient recruitment and follow-ups in a community-based health education intervention trial in hypertensive patients in Liaobu Town, Dongguan City, China.**

**Figure 2:**
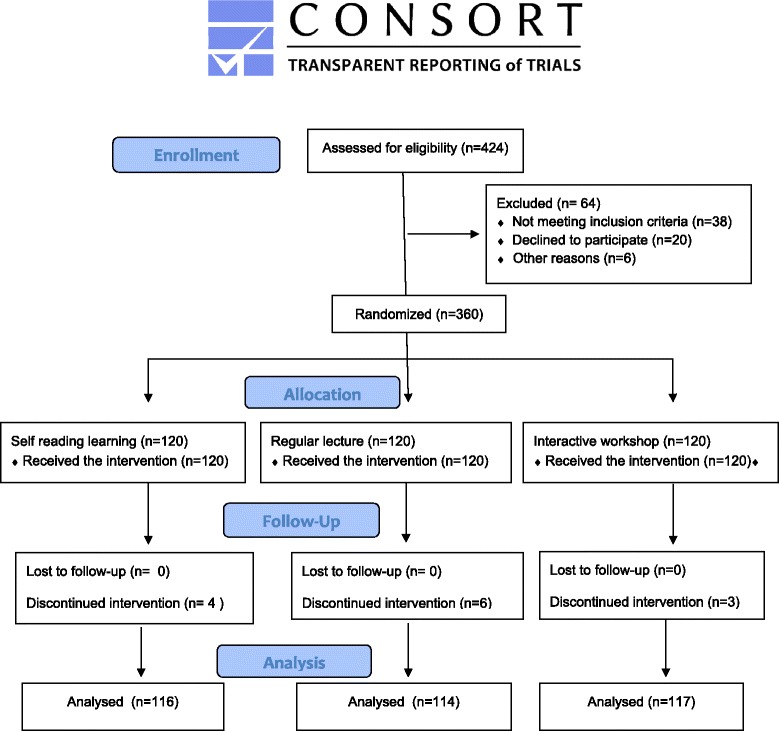
**CONSORT flow diagram of participant allocation, follow-up and analysis.**

The health education on hypertension intervention lasted two years from September 2011 to October 2013, and the study flowchart is presented in Figure 1. The “health education on hypertension syllabus” was developed by cardiovascular experts. The health education syllabus is comprised of 5 chapters with 60 sections, including hypertension-related knowledge, healthy diet, regular physical exercise, alcohol drink and cigarette smoking cessation, and adherence to anti-hypertensive medications. The learning materials were disseminated through the three different health education strategies. The numbers of drop-outs were 4, 6 and 3 in groups 1, 2, and 3, respectively, leaving 116, 114 and 117 subjects in the three groups, respectively, at the end of the study intervention for assessing the intervention effects Figure 2.

### Measurements of intervention effects

The primary outcome was the change in the proportion of subjects with normalized BP after the 2-y health education intervention. Other outcomes included the changes in hypertension-related knowledge, lifestyle, anthropometric, biochemical (serum lipids) and clinical parameters.

Hypertension related knowledge was scored based on participants’ responses to questions (Table 1). Self-reported regular use of medications for hypertension refers to strict adherence to medications following the medical instructions - defined as “the number of missed medications less than 3 times in a month”. The type, dose and number of BP lowering drugs followed the regular prescriptions by the general practitioners in the community health service centre, and the details were not recorded. Lifestyle modifications were assessed on self-reported salt intake, physical activity, smoking and alcohol use, according to the Chinese guidelines for prevention and treatment of hypertension [15]. Appropriate salt intake was defined as a salt intake of no more than 6 g per day, as estimated from monthly home consumption of salts divided by the product of 30 days multiplied by the number of individuals in the household. Regular physical activity was defined as moderate exercise lasting no less than 30 minutes, >=3 times per week. “Current smokers” were defined as those who smoked at least one pack of cigarette per month over the last 6 months. “Alcohol drinkers” were defined as those who drank alcohol at least once per week over the last 6 months.

**Table 1:** **Score criteria for questions on hypertension knowledge**

**Question**	**Option**	**Score**
**Single choice questions**		
1. Diagnostic criteria of hypertension	① 140/90 mmHg; ② 145/95 mmHg; ③ Don’t know/not sure	Correct answer 1; Wrong answer 0; Don't know/not sure 0
2. Hypertension grading	① One; ② Two; ③ Three; ④ Don’t know/not sure	
3. Benefits of regular physical activity	① Prevent hypertension; ② Can’ t prevent hypertension; ③ Don’t know/not sure	
4. Does hypertension need long duration (lifetime) of treatment?	① Yes; ② No; ③ Don’t know/not sure	
5. The upper limit of salt intake?	① No more than 2 grams a day; ② No more than 4 grams a day; ③ No more than 6 grams a day; ④ Don’t know/not sure	
6. The minimal rest time before blood pressure measurement?	① 2–3 minutes; ② 5–15 minutes; ③ Don’t know/not sure	
**Multiple choice questions**		
7. Common symptoms of hypertension	① Dizziness; ② Headache; ③ Palpitation; ④ numbness and tingling in limbs, insomnia or other symptoms; ⑤ All the above answers were correct; ⑥ Don’t know/not sure	Correct answer 1; Don’t know/not sure=0; Other answers 0.5
8. Major risk factors of hypertension	① Overweight or obesity; ② Heredity; ③ Smoking, drinking; ④ Excessive salt intake; ⑤ All the above answers were correct; ⑥ Don’t know/not sure	
9. Cardiovascular complications of hypertension if blood pressure is not controlled	① Cerebral infarction; ② Cerebral hemorrhage; ③ Coronary heart disease; ④ Renal failure; ⑤ All the above answers were correct; ⑥ Don’t know/not sure	
10. Hypertension treatment	① Antihypertensive drugs; ② Diet, lifestyle modifications; ③ All the above answers were correct; ④ Don’t know/not sure	

Blood pressure (BP) was measured on the right arm using the mercury sphygmomanometer, and the average of three readings was taken. Systolic/diastolic blood pressures were classified as normal (<140/90 mmHg), grade 1 (140/90–159/99 mmHg), grade 2 (160/100–179/109 mmHg), and grade 3 (≥180/110 mmHg) hypertension [15]. BMI was calculated as weight/ height^2^ (kg/m2). Waist circumference was measured in centimeters at the topline of the hip bone using a non-extendable tape. Serum lipids levels were obtained from regular clinical tests, including fasting total cholesterol, triglycerides, LDL-cholesterol, HDL-cholesterol.

### Data analysis

The trial’s primary outcome (BP) was assessed by the general practitioners in the community health centres who were blinded to the intervention group, and research assistants recorded the data in EXCEL. A statistician who was blinded to the intervention analyzed the data. using the statistical analysis software SPSS 13.0. To compare the changes in outcomes after vs. before the intervention in study participants, paired t-test (for continuous variables) or paired Chi-square test (for dichotomous variables) was used. To compare the differences among the three intervention groups at the baseline and after the intervention, ANOVA (for continuous variables) or Chi-square test (for categorical variables) was applied. Logistic regression analysis was used to estimate the odds ratio (OR) of normalized BP (<140/90 mmHg) after the intervention adjusted for any unbalanced baseline characteristics between the study groups. The 2-sided P values <0.05 were considered statistically significant.

## Results

### Participant characteristics

There were no statistically significant differences in socio-demographic characteristics among the three health education intervention groups (Table 2). All participants were married. There were more women than men in each of the three study groups. Over 80% participants had completed education less than high school.

**Table 2:** **Socio-demographic characteristics of study participants at the baseline**

**Group (health education strategy)**	**Group 1** ^**a**^ **(reading) (N=116)**	**Group 2** ^**a**^ **(regular lecture) (N=114)**	**Group 3** ^**a**^ **(interactive lecture) (N=117)**	***P*** ^**b**^
Age (years)	53.4**±**7.9	55.9±7.8	53.8±9.5	0.066
Gender				0.508
Male	52 (44.8)	45 (39.5)	44 (37.6)	
Female	64 (55.2)	69 (60.5)	73 (62.4)	
Education				0.958
Primary school	67 (57.8)	66 (57.9)	67 (57.3)	
Middle school	28 (24.1)	25 (21.9)	30 (25.6)	
>=High school	21 (18.1)	23 (20.2)	20 (17.1)	
Monthly income, RMB				0.358
<1000	59 (50.9)	62 (54.4)	65 (55.6)	
1000-2000	34 (29.3)	40 (35.1)	34 (29.1)	
>2000	23 (19.8)	12 (10.5)	18 (15.4)	

Data presented are mean±SD or n (%). RMB = Renminbi (Chinese currency).

^a^Excluding 4, 6 and 3 subjects who were drop-outs in groups 1, 2 and 3, respectively.

^b^P values in ANOVA (for age) or Chi-square (for other variables) tests for differences among the three groups.

### The effect of health education on hypertension knowledge, adherence to medications treatment and lifestyle (Table 3)

**Table 3:** **Baseline and post-intervention hypertension knowledge score, adherence to antihypertensive medications and lifestyle variables in hypertensive patients by mode of health education on hypertension (Group 1, reading n=116; Group 2, regular lecture n=114; Group 3, interactive workshop, n=117)**

	**Group**	**Baseline**	**Post-intervention**	***P*** ^**a**^	***P*** ^**b**^	***P*** ^**c**^
Hypertension knowledge score					<0.001	<0.001
	1	3.6±1.1	5.8±1.4	<0.001		
	2	2.7±1.9	6.6±1.5	<0.001		
	3	3.4±2.2	8.6±1.4	<0.001		
Regular use of medications					0.434	0.001
	1	46 (39.7)	59 (50.9)	<0.001		
	2	36 (31.6)	91 (79.8)	<0.001		
	3	43 (36.8)	108 (92.3)	<0.001		
Appropriate salt intake					0.235	0.075
	1	89 (76.7)	99 (85.3)	0.002		
	2	78 (68.4)	94 (82.5)	<0.001		
	3	79 (67.5)	108 (92.3)	<0.001		
Regular physical activity					0.276	<0001
	1	28 (24.1)	52 (44.8)	<0.001		
	2	25 (21.9)	76 (66.7)	<0.001		
	3	36 (30.8)	97 (82.9)	<0.001		
Current smokers					0.861	0.959
	1	22 (19.0)	17 (14.7)	0.063		
	2	20 (17.5)	16 (14.0)	0.125		
	3	19 (16.2)	18 (15.4)	1.000		
Smoked cigarettes/day					0.921	0.820
	1	3.1±7.2	2.2±5.7	0.015		
	2	3.2±8.1	2.5±7.6	0.008		
	3	3.5±8.5	2.7±7.0	0.014		
Current alcohol drinkers					0.296	0.365
	1	11 (9.5)	10 (8.6)	1.000		
	2	5 (4.4)	5 (4.4)	1.000		
	3	10 (8.5)	10 (8.5)	1.000		
Alcohol consumption					0.193	0.226
	1	26.7±106.8	26.7±106.8	1.000		
	2	8.8±53.7	8.1±53.1	0.319		
	3	38.0±175.8	19.7±78.5	0.063		

Data presented are mean± SD or n (%).

^a^P values in paired t-test (for continuous variables) or paired Chi-square test (for dichotomous variables) for differences between the baseline and post-intervention.

^b^P values in ANOVA (for continuous variables) or Chi-square (for dichotomous variables) test for differences among the three groups at the baseline survey.

^c^P value in ANOVA (for continuous variables) or Chi-square (for dichotomous variables) test for differences among the three groups after the intervention.

At the baseline, most participants (about 2/3) did not regularly take BP lowering medications. After the 2-year health education intervention, there were statistically significant increases in hypertension-related knowledge scores in all the three intervention groups. However, the increase was significantly greater in the interactive education workshop group 3 (mean score increased from 3.4 to 8.6) than in the regular lecture group 2 (mean score increased from 2.7 to 6.6) or self-learning reading group 1 (mean score increased from 3.6 to 5.8). Regular use of medications for hypertension and regular physical activity were significantly more frequent after the intervention in all the three groups, but the improvements were progressively greater from group 1 to group 2 to Group 3. The percentages of current smokers and alcohol drinkers did not change significantly in all the three health education intervention groups, although the average numbers of cigarettes smoked per day decreased slightly after the intervention. There were no significant differences in smoking and alcohol use at both the baseline or after the intervention among the three groups.

### The effect of health education on anthropometric, clinical and biochemical parameters (Table 4)

**Table 4:** **Baseline and post-intervention anthropometric, clinical and biochemical parameters in hypertensive patients by mode of health education on hypertension (Group 1, self-learning reading; Group 2, regular lecture; Group 3, interactive workshop)**

	**Group**	**Baseline**	**Post-intervention**	***P*** ^**a**^	***P*** ^**b**^	***P*** ^**c**^
Normalized blood pressure					0.872	<0.001
	1	44 (37.9)	50 (43.1)	0.451		
	2	47 (41.2)	72 (63.2)	<0.001		
	3	47 (40.2)	101 (86.3)	<0.001		
Systolic BP, mmHg					0.003	0.014
	1	140.2±17.9	139.4±16.7	0.611		
	2	143.9±16.2	134.8±15.9	<0.001		
	3	148.7±21.5	133.7±13.6	<0.001		
Diastolic BP, mmHg					0.010	0.001
	1	86.7±11.2	85.2±10.66	0.177		
	2	85.8±10.7	80.4±11.2	<0.001		
	3	90.7±16.5	81.2±8.0	<0.001		
BMI					0.111	0.001
	1	26.2±4.0	25.9±4.1	<0.001		
	2	25.6±3.5	25.5±2.9	0.361		
	3	25.2±3.7	24.1±3.7	<0.001		
Waist circumference					0.181	0.028
	1	87.0±12.1	86.0±9.5	0.207		
	2	88.8±8.8	88.7±8.5	0.488		
	3	89.3±8.7	88.7±8.2	<0.001		
Triglycerides, mg/dL					<0.001	<0.001
	1	209.9±72.6	175.6±66.8	<0.001		
	2	136.4±63.4	137.3±49.4	0.871		
	3	153.7±70.5	149.9±81.0	0.658		
Total Cholesterol, mg/dL					0.490	0.741
	1	197.7±101.5	191.2±83.8	0.154		
	2	205.7±61.3	193.6±30.9	0.010		
	3	208.8±43.3	196.8±40.1	<0.001		
LDL-cholesterol, mg/dL					0.942	0.404
	1	102.5±34.7	104.7±33.9	0.019		
	2	103.9±30.5	104.6±20.9	0.808		
	3	103.1±28.7	100.3±29.1	<0.001		
HDL-cholesterol, mg/dL					0.016	0.126
	1	41.0±7.4	43.0±9.2	0.007		
	2	42.6±7.9	43.7±11.7	0.370		
	3	39.7±7.5	41.3±5.5	0.038		

Data presented are mean± SD or n (%).

^a^P values in paired t-tests (continuous data) or paired Chi-square tests (categorical data) for differences between the baseline and post-intervention.

^b^P values in ANOVA (for continuous variables) or Chi-square (for categorical variables) test among the three groups at the baseline survey.

^c^P values in ANOVA (for continuous variables) or Chi-square (for categorical variables) test for differences among the three groups after the intervention.

BMI decreased significantly in Group 1 (self-reading) and Group 3 (interactive education workshop), but did not change significantly in group 2 (regular lecture). Waist circumference decreased significantly in Group 3 only. There were no statistically significant differences in BMI among the three groups at the baseline. The decreases in BMI were significant in both group 1 and group 3, but not group 2. Patients in group 3 had the largest reduction in BMI after the intervention.

Both systolic and diastolic BP decreased significantly, and the proportion of normalized BP increased significantly after the intervention in both group 2 and group 3 (Table 4). In contrast, there were no significant changes in systolic and diastolic BP or the proportion of normalized BP after the intervention in group 1. The increase in the proportion of subjects with normalized BP between the baseline and post-intervention was larger in group 3 (from 40.2% to 86.3%) than in group 2 (from 41.2% to 63.2%). There was a mean reduction of 15 mmHg in systolic BP and 9.5 mmHg in diastolic BP in Group 3, larger than the mean reduction of 9.1 mmHg in systolic BP, and 5.4 mmHg in diastolic BP in group 2, respectively.

At both baseline and post-intervention, fasting serum triglycerides concentrations were higher in group 1 vs. group 2 or 3, while there were no significant differences in serum total cholesterol and LDL concentrations among the three groups (Table 4). After the intervention, serum total cholesterol concentrations decreased significantly in group 2 or 3 but not group 1. Serum triglycerides concentrations decreased significantly in group 1 only, while HDL concentrations increased significantly in both group 1 or 3 but not group 2. Fasting serum LDL concentrations increased significantly in group 1, did not change significantly in group 2, but decreased significantly in group 3 after the intervention.

As compared to self-learning reading (group 1), regular lecture (group 2) or interactive workshop education on hypertension was associated with a significantly greater likelihood of normalized BP 2-years post-intervention, even after adjusting unbalanced baseline characteristics between the three groups (Table 5). The effect size (OR) was greater for interactive workshop (adjusted OR=14.66, p<0.001) than for regular lecture (adjusted OR=2.37, p=0.008).

**Table 5:** **Adjusted ORs of normalized blood pressure in hypertensive patients after health education intervention**

**Variable**	**Normalized blood pressure (<140/90 mmHg)**		
	**OR** ^**a**^	**(95% CI)** ^**c**^	**P**
Intervention^d^			
Group 1 self-learning reading	1.00		
Group 2 regular lecture	2.37	(1.26-4.47)	0.008*
Group 3 interactive workshop	14.66	(6.59-32.62)	<0.001*
Baseline characteristic^b^			
Hypertension knowledge score	1.06	(0.81-1.38)	0.676
Systolic BP	0.58	(0.43-0.79)	0.001*
Diastolic BP	0.80	(0.59-1.07)	0.134
Serum TG	0.88	(0.66-1.17)	0.369
Serum HDL	1.15	(0.87-1.51)	0.326

^a^The odds ratios of normalized blood pressure (<140/90 mm Hg) in the final model including the intervention group and baseline characteristics unbalanced between the groups (hypertension knowledge score, systolic BP, diastolic BP, serum TG and HDL).

^b^The OR per SD increase in each continuous variable.

^c^CI=confidence interval.

^d^The reference category was “Group 1 self-learning reading”.

*P<0.05.

## Discussion

After the 2-y health education interventions, generally beneficial changes were observed in hypertension-related knowledge scores, adherence to medications, appropriate salt intake and regular physical activity among all the three educational intervention groups, but the improvements were progressively greater from group 1 (self-learning) to group 2 (regular lecture) to group 3 (interactive education workshop). However, only the last two groups (2 and 3) showed significant reductions in systolic BP, diastolic BP, and the proportion of subjects achieving normalized BP, and group 3 showed the most beneficial changes in BP. The results demonstrated an effective community-based interactive health education strategy in improving clinical outcomes in hypertensive patients.

Previous reports have demonstrated that educational interventions on disease-related knowledge may help patients to better understand their health problem and its therapy leading to beneficial changes in health behaviors and adherence to regular treatment in improving health outcomes [16-20]. Similarly, our study confirmed this finding. In addition, we found that an interactive participatory health education strategy works best, as demonstrated by the most significant improvements in hypertension-related knowledge, and the largest reductions in body mass index, BP, and serum LDL levels. In general, the improvements in clinical risk factors (e.g. LDL) were the best in the interactive education workshop group. The only exception is that the improvement in serum triglycerides was the best in the self-learning group, largely because of the unbalance at the baseline; patients in the self-learning group had much higher average serum triglycerides levels at the study entry. However, the three groups had similar serum LDL levels at the baseline. We are aware of only one recent trial on health education in the management of hypertension: Ribeiro et al. compared monthly health education workshops alone to monthly education workshops combined with family orientation through home visits [4], and found the latter strategy was associated much better changes in adherence to treatment and reductions in behavioral and clinical risk parameters. Taking together, it appears that health educational interventions could be an important tool for improving clinical outcomes in hypertensive patients.

We observed no significant changes in the proportions of smokers and alcohol drinkers in all the three health education intervention groups. One possible explanation is that most smokers were nicotine-dependence, thus it is very difficult to quit smoking. Similarly, alcohol drinkers may be alcoholic and difficult to abstain. Several reports in China have showed that nicotine or alcohol dependence is associated with middle age, low education and low income [21-25], which are the characteristics of the study population.

In a systematic review (Glynn LG 2010) [26], education interventions directed at the patients showed no net large reductins in blood pressure. In this review, several RCTs (Hennessy 2006, Hunt 2004, McKinstry 2006, Watkins 1987) [27-30] evaluated education interventions by mailed educational materials (such as booklets) simliar to the intervention Group 1 in our study, and they reported no effect or at best a relatively modest effect on hypertension control. Our study extends those observations in demonstrating that interactive education workshops are much more helpful in BP control than simple self-learning education.

Main study limitation is that the trial was not blinded to participants; blinding is impossible to patients in such health educational interventions. We did not expect this would have affected the comparisons since patients did not know whether any health education program may be helpful in BP control. We only asked the question on missed medications (adherence), but had no information on the details of medications (type, dose and number). However, the randomization would likely have balanced the patients between the education intervention groups. Self-reported data may be prone to inaccuracies. Self-reported adherence to BP lowering drugs could not be taken as actual adherence, but only as an indicator of regular use of medications. Nevertheless, we expected that such inaccuracies in self-reported data were random and would not affect the validity of the comparisons.

## Conclusion

In conclusion, interactive education workshops may be the most effective strategy in community-based health education programs for hypertensive patients in improving patients’ knowledge on hypertension and alleviating clinical risk factors for preventing hypertension-related complications.

## Abbreviations

CHSI: Community health service institution
BP: Blood pressure
HDL: High-density lipoprotein
LDL: Low-density lipoprotein
TG: Triglycerides
TC: Total cholesterol
